# Effect of chain flexibility on cell adhesion: Semi-flexible model-based analysis of cell adhesion to hydrogels

**DOI:** 10.1038/s41598-019-38951-7

**Published:** 2019-02-21

**Authors:** Jooyoung Lee, Boa Song, Ramesh Subbiah, Justin J. Chung, U Hyeok Choi, Kwideok Park, Sang-Heon Kim, Seung Ja Oh

**Affiliations:** 1 Center for Biomaterials, Korea Institute of Science and Technology, Seoul, 02792 Republic of Korea; 2 Department of Polymer Engineering, Pukyong National University, Busan, 48547 Republic of Korea; 3 Department of Biomedical Engineering, University of Science and Technology, Daejon, 34113 Republic of Korea

## Abstract

Hydrogels have been developed and applied to various biomedical applications due to their biocompatibility. However, understanding of modulation between cells to hydrogel interface is still unclear, and parameters to explain the interaction are not sophisticated enough. In this report, we studied the effect of polymer chain flexibility on cell adhesion to various hydrogel constructs of collagen and fibrin gels. Specifically, novel method of semi-flexible model-based analysis confirmed that chain flexibility mediated microstructure of the hydrogels is a critical factor for cell adhesion on their surfaces. The proposed analysis showed possibility of more accurate prediction of biocompatibility of hydrogels, and it should be considered as one of the important criteria for polymer design and selections for enhancing both biocompatibility and biofunctionality.

## Introduction

Hydrogels are networks of polymer chains swollen with water and have been used in various biomedical applications such as drug delivery and tissue engineering^1–3^. Although hydrogels have attracted much attention as biomaterials due to high water content and biocompatibility, their poor mechanical properties are limiting factors for applications that require mechanical strength. Over the past decades, many efforts have focused on enhancing the mechanical strength of hydrogels^4–7^, since biomaterials are consistently exposed to tissue microenvironment, and their mechanical properties are known to affect cellular behavior. However, dynamics and communications between cells and hydrogel interfaces are poorly understood.

Cell adhesion to biomaterials is crucial for *in vivo* biocompatibility^8^. As the importance of mechanical properties of extracellular matrix (ECM) on cellular behavior has attracted much attention, researchers have tried to analyze the role of mechanical properties of biomaterials on cell adhesion. Most studies have evaluated cell adhesion behavior by analyzing bulk stiffness of materials^8–11^. However, bulk mechanical properties do not fully define cellular behavior on biomaterials since there is scale discrepancy between cells and bulk samples. Therefore, there are high demands for defining precise mechanical parameters to understand cell to biomaterial interaction, thereby predicting both biocompatibility and functionality of biomaterials.

Natural polymers, such as collagen and fibrin, are attractive sources of biomedical applications due to their excellent biocompatibility. Most natural polymers are classified as a semi-flexible polymer, where neither models of flexible chain solutions nor rigid-rod networks apply to such systems^12^. They form viscoelastic networks, where the network mesh size is smaller than persistence length, but behave like flexible coils at long ranges. Because the micrometer scale architecture of these polymeric networks interacts with cells, their mechanical properties affect the cell adhesion behavior directly. According to the semi-flexible model, chain flexibility can be predicted by the scaling of elastic plateau modulus of polymeric networks^13^. These features cannot be captured with bulk stiffness, such as Young’s modulus, used in traditional adhesion studies.

In this report, we propose a new semi-flexible model-based analysis method for understanding of cell adhesion to hydrogels by utilizing well-characterized collagen and fibrin. To investigate the determinant factors for cell adhesion properties, three physically different collagen and fibrin constructs (2D coated-substrates, 2D bulk hydrogels, and 3D bulk hydrogels) with the variation of hydrogel concentrations from 1 mg/ml to 7 mg/ml were evaluated. Additionally, the bulk stiffness and roughness were quantified, and chain morphology was observed to define parameters for cell attachment. Finally, the results confirmed that the microstructure of hydrogels affected by chain flexibility was a crucial factor for cell adhesion. The authors believe that this was the first study on evaluating the cell adhesion onto hydrogels by the slope of elasticity using a semi-flexible model. The proposed analysis method can offer guidelines for designing hydrogels and biopolymers for enhancing cell adhesion properties.

## Materials and Methods

### Hydrogel preparation

High concentration rat tail type I collagen (Corning Inc.) was dissolved in 10X M199 medium and 1X cell medium for 1, 3, 5, and 7 mg/ml concentration. Then the pH was adjusted by 1 N NaOH solution which was filtered by 0.22 μm pore filter. Bovine fibrinogen (Sigma Aldrich) was dissolved in 1X PBS in the concentration of 1, 3, 5, and 7 mg/ml. Then it was filtered by 0.22 μm pore filter before gelation process which is taken place by 2 U/ml of thrombin (bovine thrombin; Sigma-Aldrich) solution. Then the prepared solutions were casted in 24 well-plate for 2 ml volume and incubated in 37 °C overnight. The hydrogels were carefully removed from the plate for measurement of mechanical properties.

### Mechanical properties

Rheological properties of hydrogels were measured using a stress controlled type rheometer (AR-G2, TA instruments) at 37 °C with a parallel-plate (40 mm) geometry. Amplitude sweeps were first performed to get the linear viscoelastic region. Frequency sweeps were then performed at a constant strain of 0.1% in the linear viscoelastic region to determine values of the storage and the loss modulus. The compressive modulus were measured by the universal testing machine (Model 5966, Instron Corporation).

### Hydrogel morphology and young’s modulus by atomic force microscopy

The surface topography of hydrogels was evaluated using an atomic force microscope (AFM; NanoWizard II, JPK Instruments) equipped with an inverted optical microscope (Nikon), in liquid contact mode using an HYDRA2R-50NG probe at the scan size of 40 × 40 μm^2^ to accommodate for typical surface features while maintaining a high resolution.

The young’s modulus (E) of hydrogels was measured using the micro-indentation technique. A gold-coated silicon nitride (Si_3_N_4_) cantilever (PT.SiO2.AU.SN10) functionalized with a 10 µm diameter SiO_2_ particle (spring constant = 0.01 N/m) was utilized. Indentation were carried out under a ramp size of 1 μm and a loading speed of 1 μm/s. A series of indentation forces (0.5~10 nN) were applied to achieve an indentation depth of 10–20% of the measured hydrogels height. The average young’s modulus (E) was calculated using JPK data processing software, in which the Poisson ratio was set to 0.5^14^.

### Cell culture

Human umbilical vein endothelial cells (HUVEC; Lonza Inc.) cultured in Endothelial Growth Medium-2 (EGM-2, Lonza Inc.). HUVECs were cultured in 0.5% gelatin-coated 25T-flask to expand as recommended by the company.

### Cell adhesion assay

The cell adhesion rate has measured on different concentrations of collagen and fibrin hydrogels in a 2D manner. 400 μl of each hydrogel are casted in 24 well-plate and gelated for 1 hour in 37 °C incubator. Then each well of the plate was filled with 500 μl cell suspension containing 5 × 10^4^ cells ml^−1^. After 2 hours, the adhesion was quantified by Cell Counting Kit-8 (CCK-8).

### Device preparation and gelation protocol

The device designed to make 3D channels most simply. The PDMS is poured in the aluminum mold that is designed in the right size and hardens in the 70 °C oven overnight. It has a channel that is lying through space where the hydrogel is casted. The channel diameter is 500 μm. We used a stainless tube that has 500 μm outer diameters to put through the gel casting area in the middle of the PDMS mold. Then the prepared collagen or fibrin hydrogel was casted. Then we let the hydrogel gelated in the 37  °C incubators for overnight. Then we pulled the stainless tubes out from the channel.

### Endothelial cell seeding on 3D channel

The HUVECs were harvested in passage 6–8 by trypsinizing, then seeded inside the channel with a 6 × 10^6^ cells/ml concentration. The cell-seeded device was set onto the rotator for 2 hrs with the 6 round/hr speed. The inner surface of the hollow channel was observed 24 hrs after seeding, and the images were captured using the inverted microscope (Axio Vert. A1, Zeiss) equipped with CCD camera (AxioCam ICC 1, Zeiss).

### Immunofluorescence staining

For the immunofluorescence study to measure the expression levels of CD31 from the seeded HUVEC on 2D hydrogels and 3D channel constructs. The sample was fixed with the 4% paraformaldehyde for 30 mins at room temperature. Then, it was washed with 1X PBS for 10 mins, 3 times and permeablized with 0.3% of Triton X-100. The sample was incubated in 1% BSA blocking solution for 1 hr. The primary antibody (Abcam) diluted in 1% of BSA was treated at 4 °C for overnight, and secondary antibody (Thermo Fisher Scientific) was treated for 1 hr at room temperature. After 3 times of washing in 1X PBS, the sample was treated with 4’,6’-diamidino-2-phenyl indole (DAPI, Thermo Fisher Scientific) for nucleus staining. The fluorescence-labeled sample was observed under a laser scanning confocal microscope (LSM700, Zeiss).

## Results

**Figure 1:**
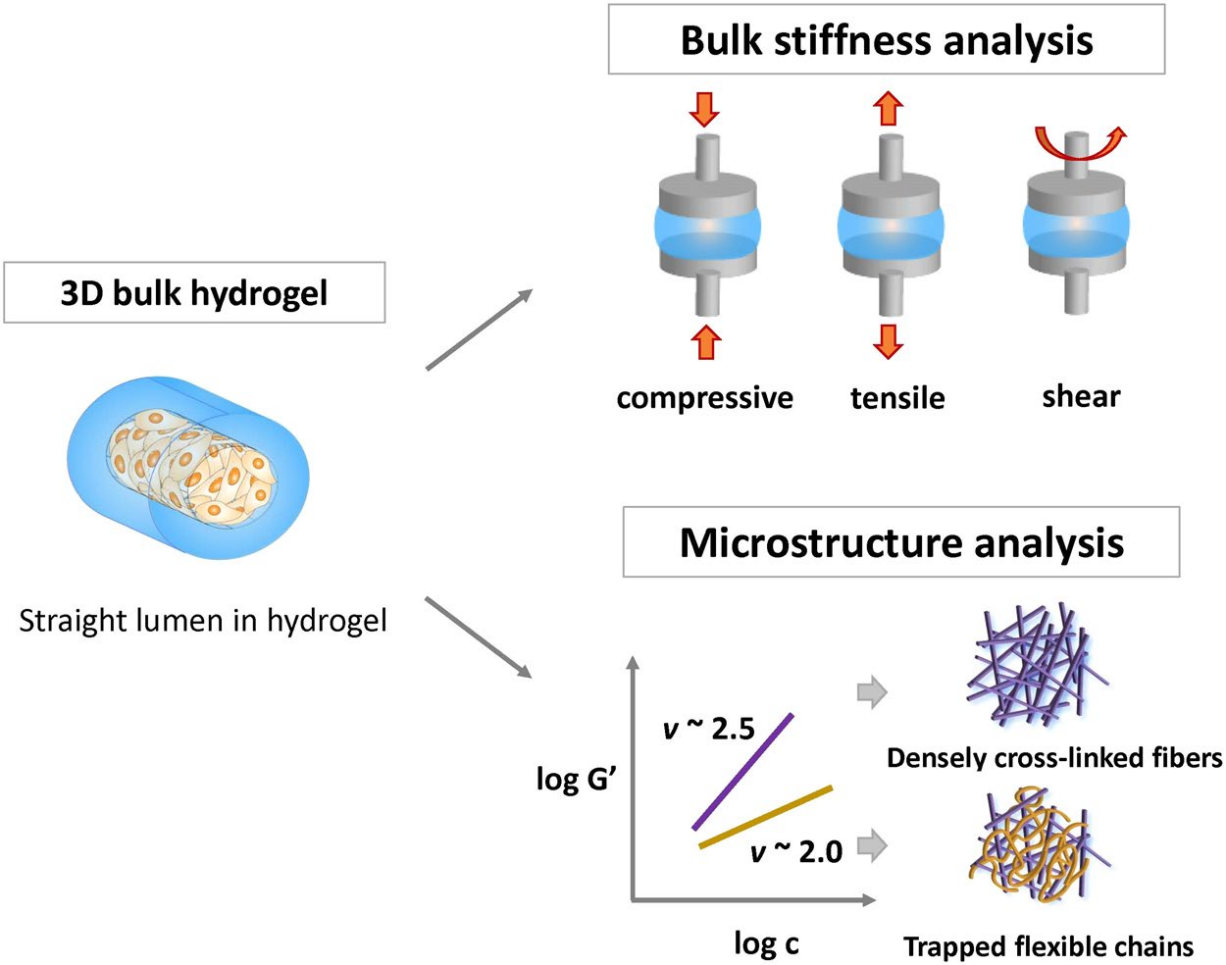
Schematic representation of methods for characterizing the physical properties of hydrogel: the bulk stiffness of hydrogel and chain flexibility in viscoelastic networks through the semi-flexible model.

**Figure 2:**
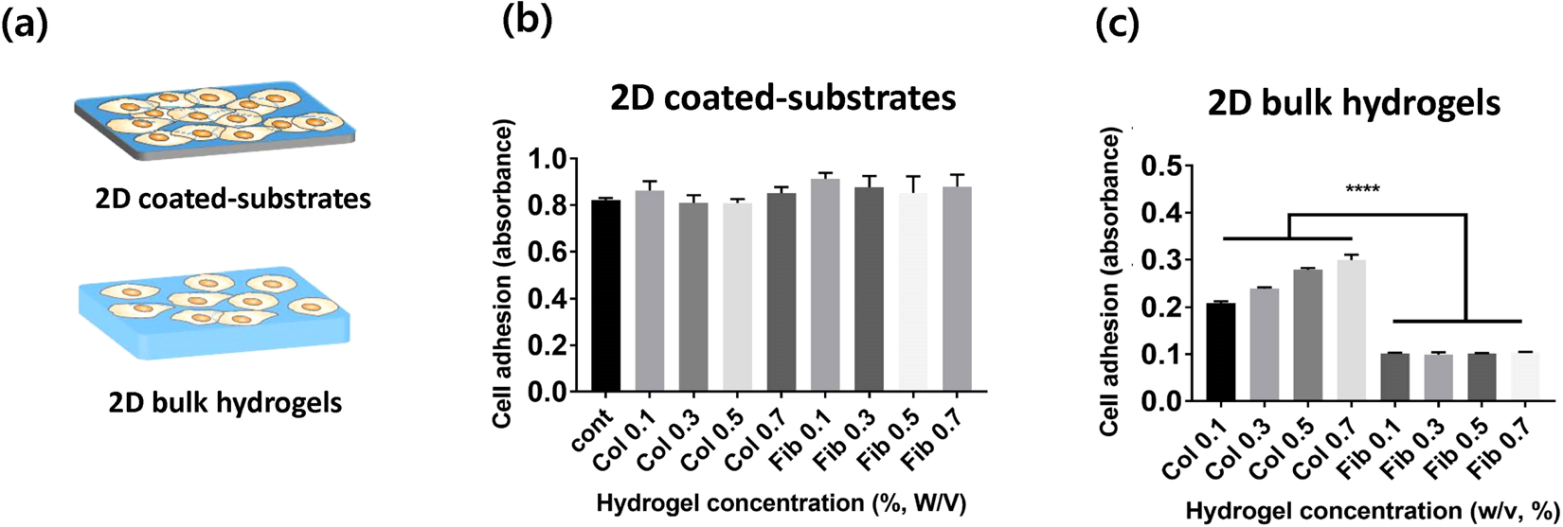
(**a**) Representation of 2 types of hydrogel constructs evaluated in this study. (**b**,**c**) Adhesion of HUVEC cells on collagen and fibrin gels onto top surfaces of 2D hydrogel-coated plates (**b**) and bulk hydrogel (**c**), which is measured by CCK-8 assay 2 hrs after seeding.

**Figure 3:**
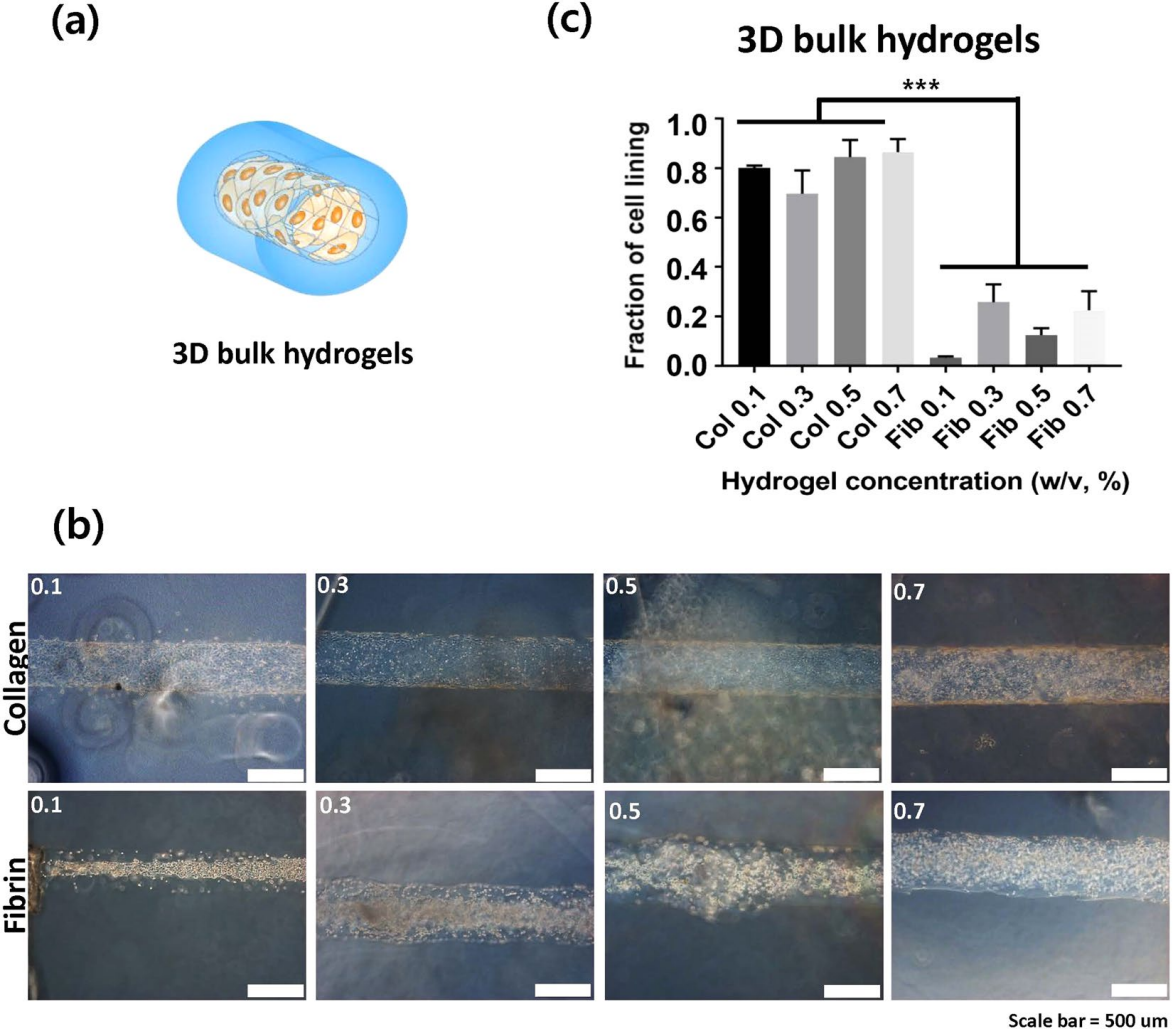
(**a**) Representation of 3D bulk hydrogel constructs (**b**) The inner surface of hollow channels in which the images were captured 24 hrs after seeding. (c) The fraction of cell lining as quantification of cell adhesion.

Figure 1 describes the scheme to explain the experimental set-up of this study and analysis methods for characterizing physical properties of hydrogels. For the experiment, various collagen and fibrin constructs (Figs 2(a) and 3(a)) were fabricated, including lumen structure to evaluate cell adhesion without gravity effect. The stiffness of the hydrogel was controlled by concentration increments of each component, then quantified by measuring the stress-strain relationship depending on the types of applied loads such as axial (tensile or compressive), and rotational (shear) stress. Chain flexibility in viscoelastic networks was evaluated based on the semi-flexible model.

### Comparison of cell adhesion onto collagen vs. fibrin gels

To understand the relationship between physical properties of hydrogels and cellular behavior on the cell-hydrogel interface, cell adhesion on 2D coated-substrate and 2D bulk hydrogels was investigated (Fig. 2(a)). For the assay, HUVEC cells were seeded on each hydrogel for 2 hrs, and the adhesion was measured by the CCK-8 assay. As shown in Fig. 2(b,c), there were no statistically significant cell adhesion differences on 2D collagen and fibrin coated-substrates. This is possibly due to cells sensing the mechanical properties of the plate rather than the properties of the hydrogels^15^. Although collagen or fibrin was coated, physical properties of the plate could dictate biochemical cues when cells have enough time for settlement. In the case of 2D bulk hydrogels, however, the cell adhesion on 2D bulk collagen gels was much higher than that on 2D fibrin gels. For varying hydrogel concentrations, the cell attachment increased systematically with the collagen concentration, while the cell adhesion on fibrin gels was independent of the hydrogel concentration.

To clarify the different rate of cell adhesion on collagen and fibrin gels, cells were seeded in the 3D lumen structure of hydrogels in order to observe cell adhesion without gravity effect. The cells were seeded on the channel walls using a rotator to aid uniform distribution in the lumen for 2 hrs, and then cell adhesion was evaluated after 24 hrs. As shown in Fig. 3, different adhesion phenotypes on collagen and fibrin gels in the 3D condition were clearer than the differences observed in 2D hydrogels. The cells were well attached to collagen gels compared to that of fibrin gels.

### Bulk mechanical properties of collagen and fibrin hydrogels

**Figure 4:**
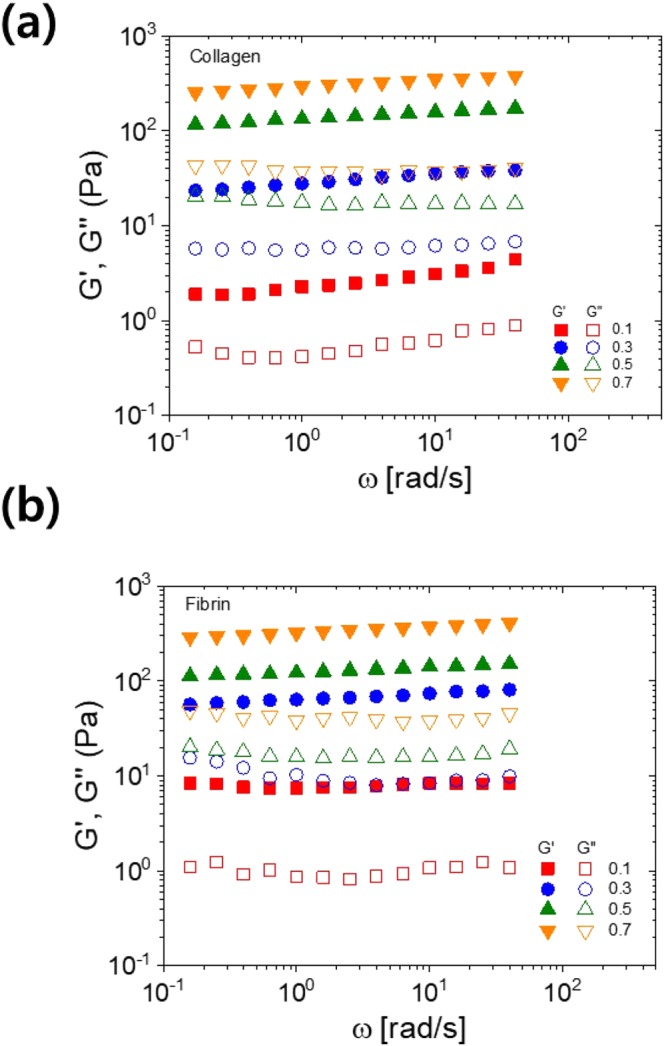
Linear viscoelastic properties of collagen and fibrin hydrogels as a function of various hydrogel concentrations: (**a**,**b**) angular frequency dependence of storage (G′, filled symbols) and loss (G″, open symbols) moduli for (**a**) collagen and (**b**) fibrin hydrogels.

To understand the underlying mechanism of different cell adhesion rate between collagen and fibrin, the physical properties of the gels were investigated. Firstly, the bulk stiffness of hydrogels was compared by measuring the linear viscoelasticity of collagen and fibrin at different concentrations; plotted in Fig. 4. Collagen and fibrin hydrogels are predominantly elastic in the entire range of frequency: G’ is independent of frequency and at least 100-fold larger than G”. This indicates that all the collagen and fibrin hydrogels from this study formed nearly perfect elastic gels on macroscopic scales^13,16^. As the concentration of collagen and fibrin increased from 1 mg/ml to 7 mg/ml, the elastic modulus gradually increased from 2 Pa to 300 Pa. Despite the different types of constituents, collagen and fibrin hydrogels showed similar elasticity values at the same concentration.

**Figure 5:**
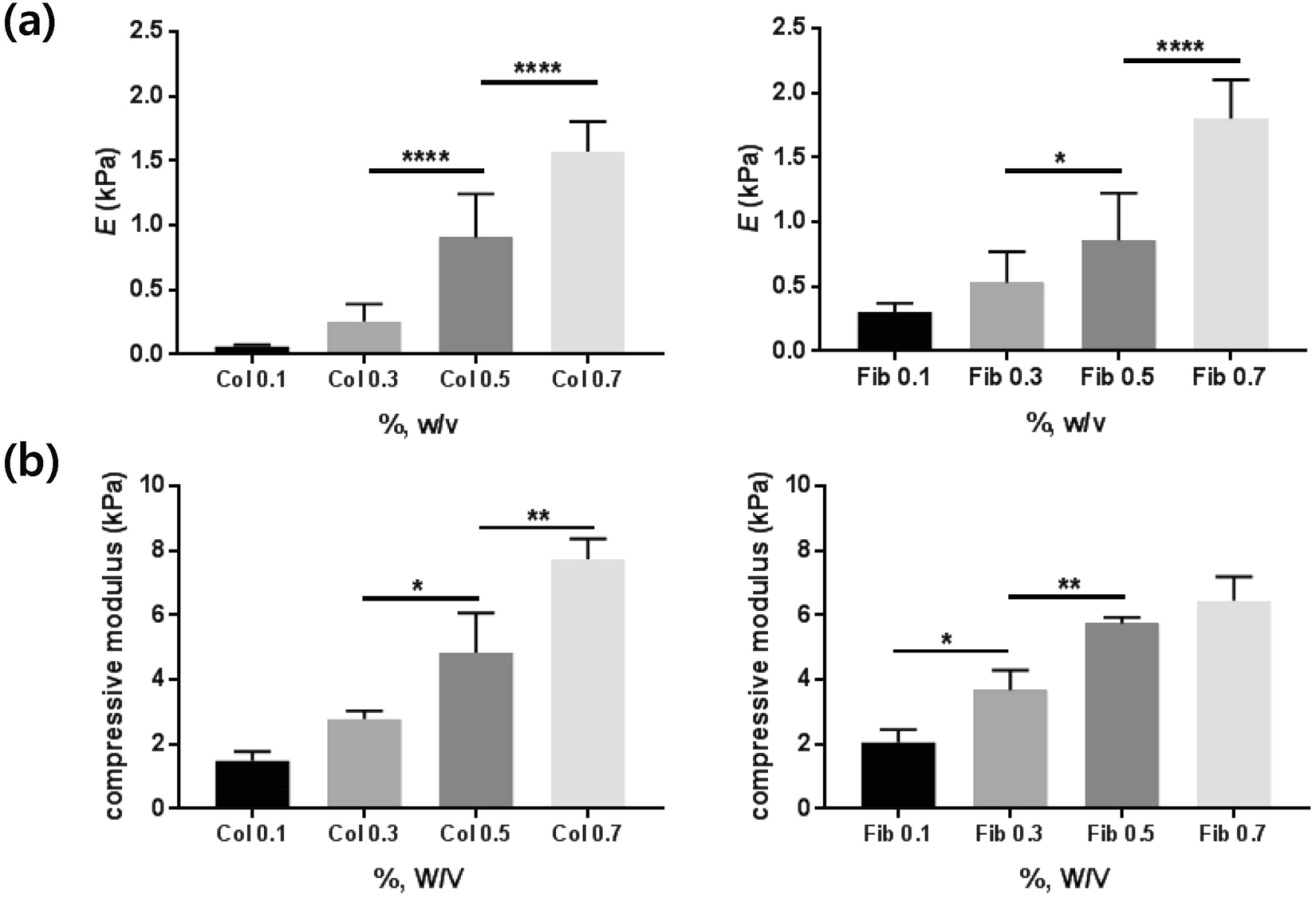
Mechanical properties of collagen and fibrin hydrogels at various concentrations: (**a**) Young’s and (**b**) compressive modulus as a function of collagen or fibrin gel concentration.

Other mechanical properties that are known to be closely related to the cell adhesion, such as Young’s modulus^10,17,18^ and compressive modulus^19,20^, were also measured and shown in Fig. 5(a,b). The tendency was similar to the results of the viscoelastic modulus: the stiffness increased as the concentration increased and showed similar values at the same concentrations regardless of the constituents. Stiffness values measured by these various methods suggested that collagen and fibrin gels show similar bulk physical properties as elastic gels on macroscopic scales. As a result, none of the methods measuring bulk physical properties could explain different cell adhesion rates between collagen and fibrin gels.

### Analysis of chain flexibility of hydrogel

**Figure 6:**
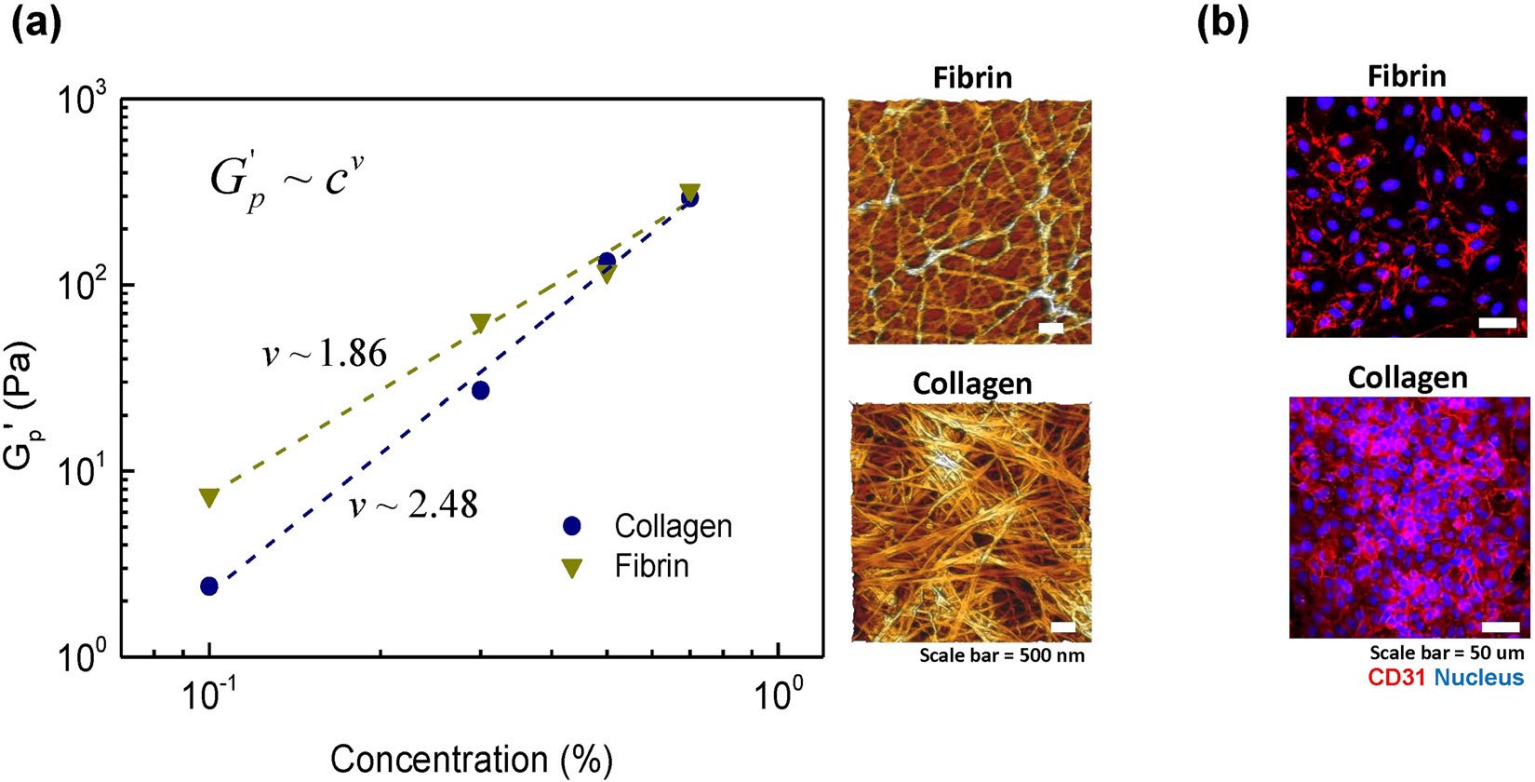
Analysis of chain flexibility of hydrogel: (**a**) power-law scaling of plateau modulus with a concentration of hydrogel for collagen and fibrin. Dash lines are the results of fits to power law equation ${G}_{p}^{\text{'}}={c}^{v}$ and microstructure of two representative samples of 0.5% concentration each in collagen and fibrin. (**b**) cell surface marker, CD31, after cells seeded for 2 hours for two representative samples of collagen and fibrin (0.5% concentration).

The concentration dependence of the elasticity in low-frequency provides another way to distinguish the physical aspects of hydrogels on microscopic scales by describing the architecture of fiber molecules and their networks^21^. As shown in Fig. 6(a), the elastic modulus in the plateau region,$${G}_{p}^{\text{'}}$$, follows a power law $${G}_{p}^{\text{'}}={c}^{v}$$$$,\,\,$$with an exponent of 2.48 and 1.86 or collagen and fibrin gels, respectively. This behavior has been explained by a semi-flexible network model which predicted $${G}_{p}^{\text{'}}={c}^{5/2}$$ for densely cross-linked gels, $${G}_{p}^{\text{'}}={c}^{11/5}$$ for semi-flexible polymers, and $${G}_{p}^{\text{'}}={c}^{2}$$ for flexible chain^12,13,22,23^. This indicates that the collagen gel structure, where $${G}_{p}^{\text{'}}$$ is more sensitive to the concentration, consists of the densely cross-linked filament, whose elastic stiffness is due to entropic origin, arising from the stretching out of individual filaments, while fibrin gel consists of relatively flexible chains, whose network elasticity is determined by chain bending, which is an enthalpic elasticity^23^.

Atomic force microscopy (AFM) was used to observe the network structures of the two gels and are shown in Fig. 6(a). Collagen gel structure displayed a fibrous network as previously reported, well-defined rod-shaped semi-flexible filaments with their straight appearance, which suggested persistence lengths of several micrometers. The fibrin gel structure, however, showed a considerably different structure to that of collagen gel: the gel structure consists of a few semi-flexible filaments, where the associated protein is much more entangled, which can be identified as dynamically trapped flexible chains.

To confirm the degree of cell attachment on the hydrogels, the cells seeded for 2 hours were fixed, and the nucleus was stained. Cell surface marker CD31 was also stained to confirm stabilized architecture of endothelium. Although bulk stiffness of collagen and fibrin gels at the same concentration was similar, cell adhesion rate difference was obvious as shown in Figs 2 and 6(b). It was found that the total amount of cells attached to collagen hydrogel was greater than that of fibrin hydrogel. It was also shown that CD31 expression of cells on collagen gel was higher than the expression on fibrin gel, indicating cells are adhering more on collagen.

Such scaling results and morphology explained the cell adhesion behavior to hydrogels. The cells were more adhesive on collagen with higher stiffness regarding the chain on the microscopic scale. In the case of fibrin with a relatively flexible chain, the cells hardly retained adhesion. Therefore, our data suggest that the microscopic stiffness of the hydrogel is a dominant factor for the degree of cell attachment.

## Discussion

Cell adhesion is fundamental in cell communication and regulation which is also critical for the development and maintenance of tissues. Specifically, robust cell adhesion to extracellular matrix (ECM) is crucial for the arrangement of multicellular components in various tissue formations. Many of previous studies have shown the effect of bulk mechanical properties of ECM on cell adhesion. Mechanical properties that are known to be closely related to the cell adhesion are Young’s modulus^10,17,18^ and compressive modulus^19,20^. Despite the use of various types of cells and substrates, most of the studies showed similar trends; substrates with higher Young’s modulus or compressive modulus were more favorable for cell adhesion. In this study, we proposed a new analysis parameter to explain cell adhesion on bulk hydrogels. Conventional methods measuring bulk stiffness or Young’s modulus were not able to determine the different cell adhesion rates on collagen and fibrin gels. We found that the microstructure of hydrogel affected by chain flexibility could be one of the factors to explain the different cell adhesion rates on hydrogels. Additionally, the microstructures of the hydrogels were defined by the slope of elasticity using a semi-flexible model.

Cells adhere to the ECM through specific anchorage points, termed focal adhesions^24,25^. Despite the cell-to-cell variations, it is generally accepted that cells on stiffer substrates have more organized cytoskeletons and stable focal adhesions^26^. Because individual focal adhesion is less than a micrometer scales, conventional stiffness measurements are not accurate procedures for the cell attachment evaluation, and more precise method of evaluating mechanical properties is required. In our experiment, bulk stiffness was not a critical factor for cell adhesion to the hydrogels.

Previous research has tried to measure the microscopic modulus of hydrogels by measuring the stress-strain relationship of a single fiber by optical tweezer or AFM^27,28^. Such results suggest that fibrin fiber is softer than that of collagen fiber. Fibrin fiber has a unique architecture which can be laterally stretched and show entropic recoil, inducing soft gel network and extraordinary extensibility, while collagen has a stiff hierarchical network of crosslinked helix fibers. Although modulus of a single fiber is critical for cell-matrix interaction, it is not represented in bulk stiffness properties of the traditional adhesion studies. Our result suggests that chain flexibility is an appropriate parameter which can reflect microscopic stiffness. Plus, it is an easier method than evaluating the modulus of a single fiber, because the linear viscoelastic modulus is very sensitive to strain exerted by cells. The method proposed in this work is most-likely applicable to other ECM hydrogels and proteins which are not evaluated in this study since most of the biopolymers have already been shown to exhibit semi-flexibility.

Collagen and fibrin have been widely used in the biomedical application as FDA approved natural polymers^29^. The analysis method proposed in this study could be practical to understand cell adhesion behavior on the surfaces of biomaterials. An accurate understanding of cell to material surface interaction will provide a significant scientific impact for the biomaterials design, although many material parameters, such as durability and mechanical strength, need to be considered for *in vivo* applications of hydrogel biomaterials.

## Conclusion

In summary, we have shown a semi-flexible model-based explanation of cell adhesion to hydrogels. Tissue stiffness measured by a shear storage modulus, or Young’s modulus has been shown to regulate cell adhesion, proliferation, and differentiation. This study now suggests that chain flexibility mediated microstructure of hydrogels is a determinant factor for cell adhesion on their surface, which was quantified by analyzing the slope of elasticity using a semi-flexible model. The authors hypothesize that this simple method can explain cell adhesion properties and offer more precise predictions in biocompatibility of polymeric biomaterials. We expect that this study provides a practical tool for the design and construction of 3D artificial tissue in various biomedical applications such as an engineered blood vessel.
